# Duration of FOLFOX Adjuvant Chemotherapy in High-Risk Stage II and Stage III Colon Cancer With Deficient Mismatch Repair

**DOI:** 10.3389/fonc.2020.579478

**Published:** 2020-12-04

**Authors:** Huabin Hu, Zehua Wu, Chao Wang, Yan Huang, Jianwei Zhang, Yue Cai, Xiaoyu Xie, Jianxia Li, Cailu Shen, Weiwei Li, Jiayu Ling, Xuehu Xu, Yanhong Deng

**Affiliations:** ^1^ Department of Gastrointestinal Surgery, The Third Affiliated Hospital of Guangzhou Medical University, Guangzhou, China; ^2^ Department of Medical Oncology, The Sixth Affiliated Hospital of Sun Yat-sen University, Guangzhou, China; ^3^ Guangdong Institute of Gastroenterology, Guangdong Provincial Key Laboratory of Colorectal and Pelvic Floor Diseases, Guangzhou, China; ^4^ Department of Pathology, The Sixth Affiliated Hospital of Sun Yat-sen University, Guangzhou, China

**Keywords:** colon cancer, adjuvant chemotherapy, deficient mismatch repair, microsatellite instability, duration of therapy

## Abstract

**Background:**

We evaluated the impact of 3 months of mFOLFOX6 adjuvant chemotherapy or surgery alone in comparison with 6 months of mFOLFOX6 on disease-free survival (DFS) in deficient mismatch repair (dMMR) colon cancer (CC) patients.

**Methods:**

This retrospective study identified a cohort of patients with high-risk stage II and III dMMR CC who underwent curative surgery between May 2011 and July 2019. DFS was compared using the Kaplan-Meier survival methods and Cox proportional hazards models. Propensity-score matching was performed to reduce imbalance in baseline characteristics.

**Results:**

A total of 242 dMMR CC patients were identified; 66 patients received 6 months of mFOLFOX6, 87 patients received 3 months of mFOLFOX6, and 89 patients were treated with surgery alone. The 3-year DFS rate was 72.8% in 3-month therapy group and 86.1% in 6-month therapy group, with a hazard ratio (HR) of 2.78 (95CI%, 1.18 to 6.47; P= 0.019). The difference in DFS between surgery alone group and 6-month therapy group was also observed but was nonsignificant (HR= 2.30, 95%CI, 0.99 to 5.38; P=0.054). The benefit of 6-month therapy in DFS compared with 3-month therapy group was pronounced for patients with stage III (HR=2.81, 95%CI, 1.03 to 7.67; P=0.044) but not for high-risk stage II patients. Propensity score matched analysis confirmed a DFS benefit in the 6-month therapy group.

**Conclusion:**

This study suggested that a 6-month duration of mFOLFOX6 adjuvant chemotherapy in dMMR CC patients may be associated with improved DFS compared with 3-month therapy, particularly in patients with stage III. The observational nature of the study implies caution should be taken in the interpretation of these results.

## Introduction

As the fourth most commonly seen cancer worldwide, colon cancer (CC) leads to 550,000 deaths each year (1). On the basis of positive findings from three phase-3 trials, 6 months of FOLFOX (fluorouracil, leucovorin, and oxaliplatin) or CAPOX (capecitabine and oxaliplatin) became the standard adjuvant therapy for patients with stage III CC (2–6). Given the neurotoxicity of oxaliplatin accumulated amid therapy that might affect patients’ daily-life activities, shorter duration of adjuvant therapy was afforded to reduce adverse effects (7). However, the non-inferiority of 3 months of adjuvant therapy with either FOLFOX or CAPOX versus 6 months was not confirmed for overall CC patients in an IDEA collaboration study. Furthermore, among the patients who received FOLFOX, results of those who received 6 months of adjuvant therapy were superior to those receiving 3 months (8, 9).

Colorectal cancer is a biologically heterogeneous disease that develops *via* 2 well described pathways of colorectal carcinogenesis, including chromosomal instability and, less commonly, microsatellite instability (MSI). MSI is a consequence of a deficient mismatch repair (dMMR) system that results in error accumulation within microsatellite region, and it occurs in approximately 15% of all colorectal cancers (10). Several studies have shown that dMMR non-metastatic CC patients were associated with a more favorable stage-adjusted prognosis compared to patients with proficient mismatch repair (pMMR) (11–14). Whether a shorter duration of adjuvant therapy for patients with dMMR would lead to any decrease in efficacy is still unclear. Thus, we evaluated the impact of 3 months of mFOLFOX6 adjuvant chemotherapy or surgery alone in comparison with 6 months of mFOLFOX6 on disease-free survival (DFS) in high-risk stage II and stage III patients with dMMR.

## Method

### Study Population

This retrospective study included all consecutive patients with histologically confirmed high-risk stage II or III CC and determined dMMR tumors who received radical surgical resection from May 2011 to July 2019 at The Sixth Affiliated Hospital of Sun Yat-Sen University. Patients with rectal cancer, incomplete curative resection (R1 or R2 resection), stage I and stage II without any high-risk factors, or adjuvant chemotherapy with fluoropyrimidine alone, CAPOX regimen (capecitabine and oxaliplatin), or duration less than 3 months were excluded. High-risk stage II CC was defined by pathologic stage T4, vascular invasion, lymphatic infiltration, perineural invasion, initial bowel obstruction, tumor perforation, or fewer than 12 excised lymph nodes (15). This study was approved by the Institutional Review Boards of The Sixth Affiliated Hospital of Sun Yat-Sen University.

### IHC Analysis of MMR Protein Expression

Formalin-fixed, paraffin-embedded tumors were stained for MLH1, MSH2, MSH6, and PMS2 proteins. Mismatch repair protein loss is defined as the absence of nuclear staining in neoplastic cells but positive nuclear staining in lymphocytes and normal adjacent colonic epithelium (16). Primary monoclonal antibodies against MLH1 (clone M1, Ventana, prediluted), MSH2 (clone G219-1129, Ventana, prediluted), MSH6 (clone 44, Ventana, prediluted), and PMS2 (clone EPR3947, Ventana, prediluted) were applied.

### MSI Testing

DNA was extracted from formalin-fixed paraffin-embedded (FFPE) tumor tissues. Comparative analysis of normal colon and tumor DNA using the five consensus monomorphic mononucleotide markers (BAT-25, BAT-26, NR-21, NR-24, NR-27) obtained through polymerase chain reaction (PCR)-based assay was adopted to assess the microsatellite instability (MSI). Specimens with at least 2 unstable markers were scored as highly unstable, while those with fewer than 2 unstable markers were stable (17).

### MMR Status Determination

MMR status was tested though the analysis of MMR protein expression by immunohistochemistry (IHC), and to be further confirmed by PCR-based MSI testing when the IHC result was undetermined. Deficient MMR phenotype tumors were defined as exhibiting the loss of expression of 1 or more MMR protein by IHC or high-level tumor DNA MSI by PCR. Tumors with discordant results between MMR protein expression and DNA MSI testing were not included in the study.

### Gene Mutation Detection

Under adequate quality-control procedures, mutation analysis was performed at the Molecular Diagnostic Laboratory of the Sixth Affiliated Hospital of Sun Yat-sen University. Genomic DNA was extracted from FFPE samples of surgery with an EZgene Tissue gDNA miniprep kit (Cat no: GD2211, Biomiga, China). KRAS (exons 2, 3, 4), NRAS (exons 2, 3, 4), BRAF (exon 15, V600E mutations), and PIK3CA (exon 9 and 20) were evaluated by bidirectional sequencing using ABI Prism 3 500 DX genetic Analyzer (Applied Biosystems, Foster City, CA).

### Treatment and follow-Up

All patients in this study underwent curative surgical resection, followed by either adjuvant chemotherapy with mFOLFOX6 regimen (oxaliplatin 85 mg/m^2^, leucovorin 400 mg/m^2^, fluorouracil 400 mg/m^2^, and fluorouracil 2400 mg/m^2^ by 48 hours continuous intravenous infusion) for 3 to 6 months or observation only. Follow-up routines consisted of physical examination, serum carcinoembryonic antigen (CEA) assay, and computed tomography scan (chest/abdominal/pelvic) every 3 to 6 months in the first 3 years and every 6 months in the following 2 years. The data were updated in December 2019.

### Propensity Score Matching

We performed propensity score matching to reduce imbalances in baseline characteristics between patients who received 3 months of mFOLFOX6 or surgery alone and those who received 6 months of mFOLFOX6. A multivariable logistic regression model was constructed to generate propensity scores. We selected covariates for inclusion in the propensity model based on factors presumed to be associated with the patient’s survival outcomes. The following baseline data were included in the model: age at diagnosis, pathologic T stage, pathologic N stage, initial bowel obstruction, vascular invasion and/or lymphatic infiltration, and perineural invasion. Patients who received 3 months of mFOLFOX6 and surgery alone were matched to those who received 6 months of mFOLFOX6 in a 1:1 ratio respectively, according to a greedy nearest-neighbor matching algorithm with no replacement. A caliper width equal to 0.2 of the standard deviation was utilized as the logit of the propensity score. We compared baseline characteristics between the propensity score-matched group using standardized differences. A standardized difference of less than 0.1 can be regarded as negligible imbalance between groups (18).

### Statistical Analysis

Categorical variables were compared by dint of the Chi-square test or Fisher’s exact test. DFS was defined as the time from surgery to the first event of local or metastatic recurrence, second primary cancer, or death from any cause. DFS curves were estimated *via* the Kaplan-Meier method and were compared by means of COX proportional hazards regression model with hazard ratios (HR), 95% confidence intervals (CI), and P values for candidate prognostic factors. Variables with P values of 0.05 or less in univariate analysis or clinically relevant were eligible for the multivariate analyses. Two-sided P values of less than 0.05 were designated as statistically significant. Apart from propensity score matching, which was implemented in R, version 3.3.2 (R Foundation), using the package Matching, all statistical analyses were performed with the 22 version of SPSS software (SPSS Inc. Chicago, IL, USA).

## Results

### Patient Characteristics

**Figure 1:**
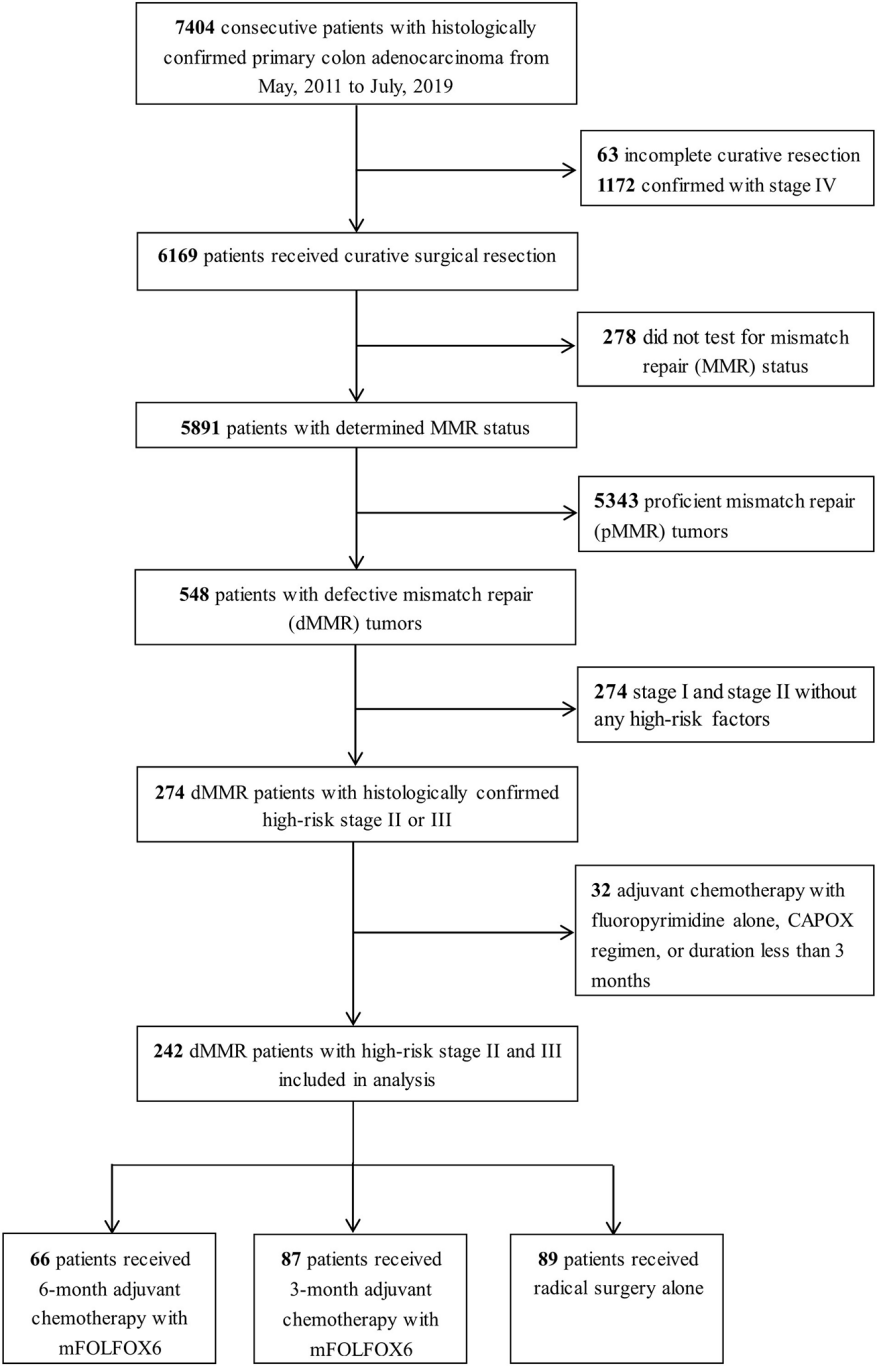
Flow diagrams of the study population.

A total of 242 patients with high-risk stage II and III dMMR CC were identified. A complete consolidated standards of reporting trials (CONSORT) diagram depicting the selection process is outlined on **Figure 1**. The median age at diagnosis was 55 (range, 22 to 88), with 64.0% of the patients being men. All patients were tested for MMR status by IHC, and 38 cases were also confirmed by PCR-MSI testing. Among 242 patients, 139 (57.4%) had lost MLH1 and PMS2 protein expression, 60 (24.8%) with MSH2 and MSH6 expression, 14 (5.8%) with MSH6 expression, and 29 (12.0%) with PMS2 expression. Additionally, 176 patients had complete data for KRAS, NRAS, BRAF, and PI3KCA status.

Overall, 153 patients received adjuvant chemotherapy with mFOLOFX6 after surgery, which consisted of 6-month therapy (27.3%, n = 66; median cycles [range] = 12 [9-12]) or 3-month therapy (36.0%, n = 87; median cycles [range] = 6 [4-7]), and 89 patients (36.7%) were treated with surgery alone. Baseline patients and tumor characteristics between treatment durations were presented in **Table 1**. Patients in the 6-month therapy group were more likely to be younger (< 65 years: 95.5%, 85.1%, 55.1%), with lower proportions of initial bowel obstruction (27.3%, 33.3%, 47.2%), but more of them proceeded to stage III (74.2%, 57.5%, 42.7%) with more positive lymph nodes examined (N1: 53.0%, 42.5%, 36.0%; N2: 21.2%, 14.9%, 6.7%) than those in the 3-month therapy and surgery alone group.

**Table 1:** Patients and tumor characteristics.

Characteristics	Missing values	All the population, n=242	6-month therapy group, n=66	3-month therapy group, n=87	Surgery alone group, n=89	*P*
		No. (%)	No. (%)	No. (%)	No. (%)	
Adjuvant therapy duration						
Median (range), weeks		–	24 (20–27)	12 (12–16)	0	
Completion no. of cycles						
Median (range)		–	12 (9–12)	6 (4–7)	0	
Age, years						<0.001
< 65		186 (76.9%)	63 (95.5%)	74 (85.1%)	49 (55.1%)	
≧65		56 (23.1%)	3 (5.5%)	13 (14.9%)	40 (44.9%)	
Gender						0.331
Female		87 (36.0%)	19 (28.8%)	35 (40.2%)	33 (37.1%)	
Male		155 (64.0%)	47 (71.2%)	52 (59.8%)	56 (62.9%)	
Grade of differentiation						0.214
Well or moderately		120 (49.6%)	27 (40.9%)	44 (50.6%)	49 (55.1%)	
Poorly		122 (50.4%)	39 (59.1%)	43 (49.4%)	40 (44.9%)	
Primary tumor site						0.259
Left (splenic flexure, descending colon, and sigmoid colon)		94 (38.8%)	21(31.8%)	39 (44.8%)	34 (38.2%)	
Right (cecum, ascending colon, hepatic flexure, and transverse colon)		148 (61.2%)	45 (68.2%)	48 (55.2%)	55 (61.8%)	
Initial bowel obstruction						0.028
No		153 (63.2%)	48 (72.7%)	58 (66.7%)	47 (52.8%)	
Yes		89 (36.8%)	18 (27.3%)	29 (33.3%)	42 (47.2%)	
Vascular invasion and/or lymphatic infiltration						0.282
No		186 (76.9%)	55 (83.3%)	63 (72.4%)	68 (76.4%)	
Yes		56 (23.1%)	11(16.7%)	24 (27.6%)	21 (23.6%)	
Perineural invasion						0.621
No		220 (90.9%)	61(92.4%)	77 (88.5%)	82 (92.1%)	
Yes		22 (9.1%)	5 (7.6%)	10 (11.5%)	7 (7.9%)	
No. of lymph nodes excised						0.503
< 12		19 (7.9%)	3 (4.5%)	8 (9.2%)	8 (9.0%)	
≧12		223 (92.1%)	63 (95.5%)	79 (90.8%)	81(91.0%)	
Pathologic T stage						0.411
T1–T3		193 (79.8%)	51 (77.3%)	67 (77.0%)	75 (84.3%)	
T4		49 (20.2%)	15 (22.7%)	20 (23.0%)	14 (15.7%)	
Pathologic N stage						0.002
N0		105 (43.4%)	17 (25.8%)	37 (42.5%)	51 (57.3%)	
N1		104 (43.0%)	35 (53.0%)	37 (42.5%)	32 (36.0%)	
N2		33 (13.6%)	14 (21.2%)	13 (14.9%)	6 (6.7%)	
Pathologic TNM stage						<0.001
High-risk stage II		105 (43.4%)	17 (25.8%)	37 (42.5%)	51 (57.3%)	
Stage III		137 (56.6%)	49 (74.2%)	50 (57.5%)	38 (42.7%)	
KRAS mutation						0.602
No		109 (61.2%)	30 (57.7%)	43 (59.7%)	36 (66.7%)	
Yes		69 (38.8%)	22 (42.3%)	29 (40.3%)	18 (33.3%)	
Missing values	64					
NRAS mutation						
No		174 (98.9%)	51 (100%)	71 (100%)	52 (96.3%)	0.102
Yes		2 (1.1%)	0	0	2 (3.7%)	
Missing values	64					
BRAF mutation						0.073
No		161 (90.4%)	50 (96.2%)	66 (91.7%)	45 (83.3%)	
Yes		17 (9.6%)	2 (3.8%)	6 (8.3%)	9 (16.7%)	
Missing values	66					
PIK3CA mutation						0.765
No		148 (84.1%)	44 (86.3%)	58 (81.7%)	46 (85.2%)	
Yes		28 (15.9%)	7 (13.7%)	13 (18.3%)	8 (14.8%)	
Missing values	66					

### Association of Adjuvant Chemotherapy Duration and Disease-Free Survival

For the overall cohort at the time of data cutoff, the median follow-up was 21.9 months. There were 17 DFS events in patients with high-risk stage II and 29 in stage III disease that led to a 3-year DFS rate of 79.5% (95%CI, 70.1% to 88.9%) and 73.4% (95%CI, 64.2% to 82.6%) respectively.

**Figure 2:**
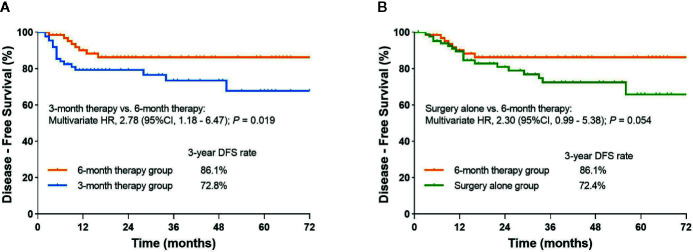
**(A)** Kaplan-Meier curve of disease-free survival comparing 6-month therapy group and 3-month therapy group; **(B)** Kaplan-Meier curve of disease-free survival comparing 6-month therapy group and surgery alone group.

The 3-year DFS rate was 72.8% (95%CI, 61.0% to 84.6%) for patients who received 3 months of mFOLFOX6 therapy and 86.1% (95%CI, 77.1% to 95.1%) for patients who received 6 months of therapy, along with an estimated multivariate HR of 2.78 (95CI%, 1.18 to 6.47; P= 0.019). For the patients treated with surgery alone, the 3-year DFS rate was 72.4% (95%CI, 60.8% to 84.0%). The multivariate HR for DFS compared with 6-month therapy was 2.30 (95%CI, 0.99 to 5.38; P=0.054) (**Figure 2**, **Table 2**, and **Supplementary Table S1**).

**Table 2:** Univariate and multivariate Cox proportional hazards regression model for disease-free survival.

Variable	No. patients	No. events	Univariate analysis		Multivariate analysis	
			HR (95% CI)	*P*	HR (95% CI)	*P*
Total	242	46				
Age						
<65 years	186	33	1	0.360		
≧65 years	56	13	1.35 (0.71–2.56)			
Gender						
Female	87	16	1	0.900		
Male	155	30	0.96 (0.52–1.76)			
Grade of differentiation						
Well or moderately	120	23	1	0.970		
Poorly	122	23	1.01 (0.57–1.80)			
Primary tumor site						
Left	94	23	1	0.170		
Right	148	23	0.67 (0.37–1.19)			
Initial bowel obstruction						
No	153	29	1	0.580		
Yes	89	17	0.84 (0.46–1.54)			
Vascular invasion and/or lymphatic infiltration						
No	186	31	1	0.014	1	0.059
Yes	56	15	2.16 (1.17–4.01)		1.89 (0.98–3.68)	
Perineural invasion						
No	220	37	1	0.001	1	0.004
Yes	22	9	3.37 (1.62–6.99)		3.26 (1.47–7.24)	
No. of lymph nodes excised						
≧12	223	38	1	0.002	1	<0.001
< 12	19	8	3.33 (1.55–7.14)		5.09 (2.23–11.62)	
Pathologic T stage						
T1-T3	193	29	1	<0.001	1	<0.001
T4	49	17	3.07 (1.68–5.61)		3.39 (1.83–6.30)	
Pathologic N stage						
N0	105	17	1	0.220	1	0.081
N1-2	137	29	1.45 (0.80–2.64)		1.80 (0.93–3.47)	
KRAS mutation						
No	109	15	1	0.278		
Yes	69	12	1.53 (0.71–3.27)			
BRAF mutation						
No	161	25	1	0.971		
Yes	17	2	0.97 (0.23–4.12)			
PIK3CA mutation						
No	148	22	1	0.315		
Yes	28	5	1.65 (0.62–4.38)			
Adjuvant chemotherapy duration						
6-month therapy group	66	8	1		1	
3-month therapy group	87	19	2.43 (1.06–5.55)	0.036	2.78 (1.18–6.47)	0.019
Surgery alone group	89	19	1.95 (0.85–4.46)	0.113	2.30 (0.99–5.38)	0.054

CI, confidence interval; HR, hazard ratio.

**Figure 3:**
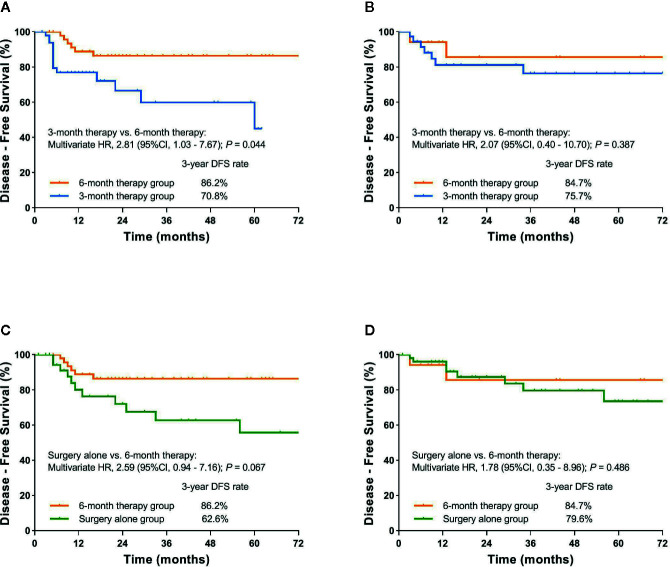
**(A)** Kaplan-Meier curve of disease-free survival comparing 6-month therapy group and 3-month therapy group for patients with pathologic stage III; **(B)** Kaplan-Meier curve of disease-free survival comparing 6-month therapy group and 3-month therapy group for patients with high-risk stage II; **(C)** Kaplan-Meier curve of disease-free survival comparing 6-month therapy group and surgery alone group for patients with pathologic stage III; **(D)** Kaplan-Meier curve of disease-free survival comparing 6-month therapy group and surgery alone group for patients with high-risk stage II.

Subgroup analysis demonstrated 3-year DFS rate for patients with stage III was 70.8% in the 3-month therapy group and 86.2% in the 6-month therapy group (HR=2.81, 95%CI, 1.03 to 7.67; P=0.044). In high-risk stage II subgroup analysis, no significant effect of adjuvant chemotherapy duration on DFS was observed in the 3-month therapy group compared with the 6-month therapy group (**Figure 3** and **Supplementary Table S1**).

### Disease-Free Survival After Propensity Score Matching

At 1:1 propensity score matching, 51 patients who received 3-month therapy and 35 patients treated with surgery alone were matched to the patients who received 6-month therapy respectively. As shown in **Table 3**, after propensity score matching, standardized differences for all included covariates among patients who received 6-month, 3-month therapy, and surgery alone were all less than 0.1, indicating a well-balanced covariate distribution after matching.

**Table 3:** Selected baseline characteristics before and after propensity score matching.

Characteristics	No. (%)				No. (%)			
	Before matching				After matching			
	6-month therapy group, n=66	3-month therapy group, n=87	*P*	Standardized difference	6-month therapy group, n=51	3-month therapy group, n=51	*P*	Standardized difference
Age, years								
Median (range)	49 (22–78)	50 (23–77)	0.337	0.190	50 (22–78)	48 (26–76)	0.269	0.030
< 65	63 (95.5%)	74 (85.1%)			49 (96.1%)	45 (88.2%)		
≧65	3 (5.5%)	13 (14.9%)			2 (3.9%)	6 (11.8%)		
Pathologic T stage			1.000	0.006			1.000	<0.001
T1-3	51 (77.3%)	67 (77.0%)			38 (74.5%)	38 (74.5%)		
T4	15 (22.7%)	20 (23.0%)			13 (25.5%)	13 (25.5%)		
Pathologic N stage			0.040	0.357			1.000	0.042
N0	17(25.8%)	37(42.5%)			16 (31.4%)	15 (29.4%)		
N1-2	49 (74.2%)	50 (57.5%)			35 (68.6%)	36 (70.6%)		
Initial bowel obstruction			0.481	0.131			1.000	0.042
No	48 (72.7%)	58 (66.7%)			36 (70.6%)	35 (68.6%)		
Yes	18 (27.3%)	29 (33.3%)			15 (29.4%)	16 (31.4%)		
Vascular invasion and/or lymphatic infiltration			0.124	0.264			1.000	0.046
No	55 (83.3%)	63 (72.4%)			40 (78.4%)	39 (76.5%)		
Yes	11 (16.7%)	24 (27.6%)			11 (21.6%)	12 (23.5%)		
Perineural invasion			0.585	0.133			1.000	0.068
No	61 (92.4%)	77 (88.5%)			47 (92.2%)	46 (90.2%)		
Yes	5 (7.6%)	10 (11.5%)			4 (7.8%)	5 (9.8%)		
**Characteristics**	**No. (%)**				**No. (%)**			
	**Before matching**				**After matching**			
	**6-month therapy group, n=66**	**Surgery alone group, n=89**	***P***	**Standardized difference**	**6-month therapy group, n=35**	**Surgery alone group, n=35**	***P***	**Standardized difference**
Age, years								
Median (range)	49 (22–78)	63 (22–88)	<0.001	1.053	49 (22–78)	56 (23–76)	1.000	<0.001
< 65	63 (95.5%)	49 (55.1%)			32 (91.4%)	32 (91.4%)		
≧65	3 (5.5%)	40 (44.9%)			3 (8.6%)	3 (8.6%)		
Pathologic T stage			0.302	0.177			1.000	<0.001
T1-3	51 (77.3%)	75 (84.3%)			27 (77.1%)	27 (77.1%)		
T4	15 (22.7%)	14 (15.7%)			8 (22.9%)	8 (22.9%)		
Pathologic N stage			<0.001	0.671			1.000	<0.001
N0	17 (25.8%)	51 (57.3%)			14 (40.0%)	14 (40.0%)		
N1-2	49 (74.2%)	38 (42.7%)			21 (60.0%)	21 (60.0%)		
Initial bowel obstruction			0.013	0.418			1.000	0.063
No	48 (72.7%)	47 (52.8%)			25 (71.4%)	26 (74.3%)		
Yes	18 (27.3%)	42 (47.2%)			10 (28.6%)	9 (25.7%)		
Vascular invasion and/or lymphatic infiltration			0.322	0.172			1.000	0.094
No	55 (83.3%)	68 (76.4%)			32 (91.4%)	31 (88.6%)		
Yes	11 (16.7%)	21 (23.6%)			3 (8.6%)	4 (11.4%)		
Perineural invasion			1.000	0.011			1.000	<0.001
No	61 (92.4%)	82 (92.1%)			32 (91.4%)	32 (91.4%)		
Yes	5 (7.6%)	7 (7.9%)			3 (8.6%)	3 (8.6%)		

**Figure 4:**
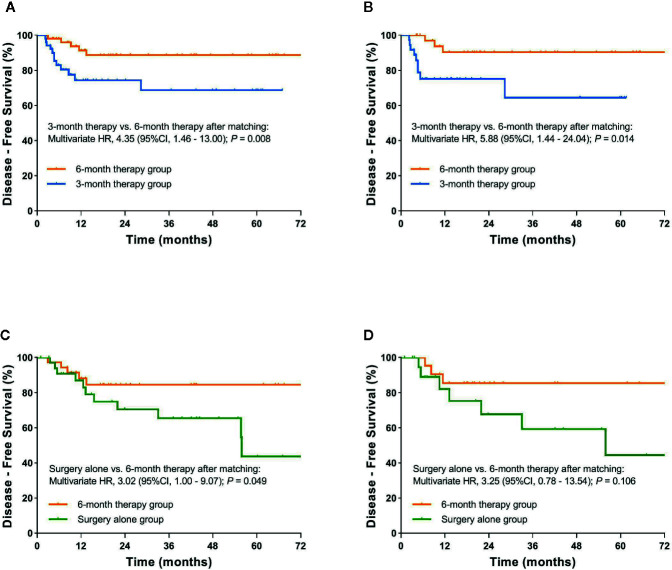
**(A)** Kaplan-Meier curve of disease-free survival comparing 3-month therapy group and 6-month therapy group after propensity score matching; **(B)** Kaplan-Meier curve of disease-free survival comparing 3-month therapy group and 6-month therapy group for patients with stage III after propensity score matching; **(C)** Kaplan-Meier curve of disease-free survival comparing surgery alone group and 6-month therapy group after propensity score matching; **(D)** Kaplan-Meier curve of disease-free survival comparing surgery alone and 6-month therapy group for patients with stage III after propensity score matching.

After matching, a significant difference in DFS in favor of the 6-month therapy group compared to the 3-month therapy group was observed. The 3-year DFS rate was 88.7% (95%CI, 79.3% to 98.1%) and 68.7% (95%CI, 52.2% to 85.2%) respectively, plus an estimated multivariate HR of 4.35 (95CI%, 1.46 to 13.00; P= 0.008). In subgroup analysis, we identified a benefit on DFS of the 6-month adjuvant chemotherapy compared with the 3-month therapy for stage III patients (3-year DFS rate: 90.3% vs. 64.5%; HR=5.88, 95%CI, 1.44 to 24.04; P=0.014) (**Figure 4**). In contrast, there was still no significant difference in DFS between the two groups for high-risk stage II patients (**Supplementary Table S2**). Marginally significant difference in DFS between the 6-month therapy group and the surgery alone group was observed in the multivariable analysis (HR=3.02, 95%CI, 1.00 to 9.07; P=0.049). The 3-year DFS rate was 84.6% (95%CI, 72.1% to 97.1%) and 65.4% (95%CI, 46.6% to 84.2%) respectively (**Figure 4** and **Supplementary Table S3**).

## Discussion

To our knowledge, this is the first study to explore the effect of the duration of mFOLFOX6 adjuvant chemotherapy on DFS in high-risk stage II and III dMMR CC patients, and it showed a statistically significant benefit in DFS in 6-month therapy group compared with the 3-month therapy group, particularly in patients with pathologic stage III.

Current guidelines recommend that all dMMR stage II CC patients, regardless of high-risk factors, should not receive 5-FU-based adjuvant therapy (11, 19–25). For dMMR stage III CC patients, adjuvant therapy with CAPOX or FOLFOX regimen is recommended, but the role of the MMR status as a predictive biomarker is still not completely clear (23, 24, 26–28). IDEA collaboration and IDEA France study have failed to demonstrate the non-inferiority of 3-month FOLFOX to 6-month FOLFOX in stage III CC patients; however, these analyses did not perform a subgroup analysis of patients’ dMMR status (8, 9). In keeping with the IDEA study, we observed that the 6-month duration of mFOLFOX6 adjuvant therapy may provide an additional DFS benefit compared with 3-month duration for dMMR stage III CC patients. In this subgroup of high-risk stage II CC patients, there were no significant differences in DFS among three groups, which implied that dMMR high-risk stage II CC patients did not significantly benefit from the FOLFOX adjuvant therapy however long the therapy duration was. The good prognosis of dMMR stage II CC patients, compared with stage III patients, might comparatively benefit less from the adjuvant therapy.

Some previous data suggested that the effect of 5-FU chemotherapy was dependent on the MMR status, and that dMMR CC patients might not benefit from 5-FU monotherapy compared to patients with pMMR CC (11, 19, 23, 29). A possible biological explanation is that in the absence of a functional MMR system, repair may only occur through the “base excision repair” system, a process that is less affected by the dNTP disequilibrium induced by 5-FU (30). However, prolonged treatment with 5-FU leads to the accumulation of DNA lesions that are targeted by another repair pathway (31), and oxaliplatin forms DNA adducts that result in the distortion of secondary DNA structure that is poorly recognized by MMR complexes (32, 33). These might explain why 6-month FOLFOX adjuvant therapy was superior to 3-month therapy in this present study.

Perineural invasion, less than 12 lymph nodes excised, and T4 are independently associated with the decreased DFS in the present analyses. There was no significant difference in DFS among different therapy duration groups in these subgroups of patients, which might be due to the limited number of the cases (data are not shown). These assessments of high-risk factors in daily practice should be discussed because they provide potentially important prognostic information for dMMR CC patients and will help to tailor adjuvant therapy.

Molecular testing (RAS, BRAF) is currently a routine part of clinical practice in colorectal cancer. However, for stage II and III CC patients, the prognostic role of these markers is controversial, particularly among dMMR patients. In a pooled analysis of PETACC-8 and N0147, and a post hoc analysis of the PETACC-8, both included resected stage III colon cancer patients receiving adjuvant FOLFOX, BRAF or KRAS mutations are independently associated with the decreased DFS in patients with pMMR, but not dMMR tumors (34, 35). These findings could explain why there was no difference of DFS in patients with KRAS, BRAF, or PIK3CA mutation tumors as compared with wild-type patients in our study.

There were some limitations of our study. First, this was a single-center retrospective study that caused the imbalances in baseline characteristics among the three groups. Fewer patients in the 3-month therapy group and the surgery alone group were in stage III than those in the 6-month therapy group. Propensity score matching was conducted to mitigate the potential bias caused by confounding covariates. The differences in DFS between the 6-month therapy group and the 3-month therapy group were consistent and robust before and after matching. Nevertheless, high-quality randomized controlled clinical trials or subgroup analyses based on a large sample size from IDEA collaboration study are demanded to confirm the optimal duration of chemotherapy for patients with stage III dMMR CC. Second, the duration of adjuvant therapy was left to investigators’ discretion in this observation, which was mainly based on not just the disease characteristics but also patient’s age and preference. Before matching, the median age of patients in the surgery alone group was significantly older than that of patients in the 3-month therapy group and the 6-month therapy group, that is, 63, 50, and 49 years old respectively. MOSAIC and NSABP C-07 study revealed no statistically significant survival benefit for the addition of oxaliplatin to fluorouracil with leucovorin as adjuvant treatment for patients older than 70 (36, 37). In addition, patients with dMMR tumors may not benefit from adjuvant chemotherapy with 5-FU alone. For stage III patients in the surgery alone group, 26.3% of them were older than 70. These may be a potential reason why 38 stage III patients did not receive any adjuvant chemotherapy. Third, dMMR in colon cancer is most commonly caused by epigenetic inactivation of MLH1 by promoter hypermethylation in a setting of CpG island methylator phenotype (CIMP) in sporadic tumors (approximately 75%) (38, 39), and the remainder of dMMR tumors are associated with germline mutations (40). A study based on a large database from randomized trials in colon cancer stage III patients suggested that the dMMR tumors with suspected germline mutations were associated with improved DFS after 5-FU-based adjuvant treatment compared with sporadic tumors where no benefit was observed (24). The absence of the family history information and methylation status of MLH1 of our cohort made it difficult to analyze the mechanism of MMR deficiency.

In conclusion, this study suggests that 6-month duration of mFOLFOX6 adjuvant chemotherapy in dMMR CC patients may be associated with improved DFS compared with 3-month therapy, particularly in stage III patients. However, these results are limited by the presence of potential unmeasured confounding in this retrospective study and require further investigations for confirmation.

## Data Availability Statement

The original contributions presented in the study are included in the article/**Supplementary Material**. Further inquiries can be directed to the corresponding authors.

## Ethics Statement

This study was approved by the Institutional Review Boards of The Sixth Affiliated Hospital of Sun Yat-Sen University. Written informed consent for participation was not required for this study in accordance with the national legislation and the institutional requirements.

## Author Contributions

HH, ZW, and YH carried out the studies, participated in collecting data, and drafted the manuscript. CW, JZ, YC, JXL, XYX, CS, WL, and JYL performed the statistical analysis and participated in its design. XHX and YD participated in acquisition, analysis, or interpretation of data and drafted the manuscript. All authors contributed to the article and approved the submitted version.

## Funding

This work was supported by Science and Technology Planning Project of Guangzhou (Grant No. 201904010394) and Science and Technology Program of Guangzhou (Grant No. 201803010073).

## Conflict of Interest

The authors declare that the research was conducted in the absence of any commercial or financial relationships that could be construed as a potential conflict of interest.
