# Supplementary material for: Duration of FOLFOX Adjuvant Chemotherapy in High-Risk Stage II and Stage III Colon Cancer With Deficient Mismatch Repair
Source: Front Oncol. 2020 Dec 4;10:579478. doi: 10.3389/fonc.2020.579478 (PMC7747753; doi:10.3389/fonc.2020.579478)
Supplement: Supplementary file 1 [file Table_1.docx]

| Supplementary Table S1. Analysis of disease-free survival (DFS) according to adjuvant chemotherapy duration and pathologic TNM stage in univariate and multivariate models. | | | | | |
| --- | --- | --- | --- | --- | --- |
| **Variable** | **No. of patients (3-year DFS rate, %)** | **DFS** | | | |
|  |  | **Univariate analysis** | | **Multivariate analysis ^*^** | |
|  |  | **HR (95% CI)** | ***P*** | **HR (95% CI)** | ***P*** |
| All patients |  |  |  |  |  |
| 6 - month therapy group | 66 (86.1) | 1 |  | 1 |  |
| 3 - month therapy group | 87 (72.8) | 2.43 (1.06-5.55) | 0.036 | 2.78 (1.18-6.47) | 0.019 |
| Surgery alone group | 89 (72.4) | 1.95 (0.85-4.46) | 0.110 | 2.30 (0.99-5.38) | 0.054 |
| Stage III |  |  |  |  |  |
| 6 - month therapy group | 49 (86.2) | 1 |  | 1 |  |
| 3 - month therapy group | 50 (70.8) | 3.06 (1.14-8.19) | 0.026 | 2.81 (1.03-7.67) | 0.044 |
| Surgery alone group | 38 (62.6) | 2.71 (1.00-7.33) | 0.050 | 2.59 (0.94-7.16) | 0.067 |
| High-risk Stage II |  |  |  |  |  |
| 6 - month therapy group | 17 (84.7) | 1 |  | 1 |  |
| 3 - month therapy group | 37 (75.7) | 1.78 (0.37-8.57) | 0.480 | 2.07 (0.40-10.70) | 0.387 |
| Surgery alone group | 51 (79.6) | 1.40 (0.30-6.57) | 0.680 | 1.78 (0.35-8.96) | 0.486 |

Abbreviation: CI, confidence interval; DFS, disease-free survival; HR, hazard ratio.

* Multivariate analysis adjusted for pathologic T stage, pathologic N stage, vascular invasion and/or lymphatic infiltration, perineural invasion, no. of lymph nodes excised.
