# Supplementary material for: Duration of FOLFOX Adjuvant Chemotherapy in High-Risk Stage II and Stage III Colon Cancer With Deficient Mismatch Repair
Source: Front Oncol. 2020 Dec 4;10:579478. doi: 10.3389/fonc.2020.579478 (PMC7747753; doi:10.3389/fonc.2020.579478)
Supplement: Supplementary file 2 [file Table_2.docx]

| Supplementary Table S2. Analysis of disease-free survival (DFS) between patients in 6-month therapy group and 3-month therapy group after propensity score matching. | | | | | |
| --- | --- | --- | --- | --- | --- |
| **Variable** | **No. of patients (3-year DFS rate, %)** | **DFS** | | | |
|  |  | **Univariate analysis** | | **Multivariate analysis ^*^** | |
|  |  | **HR (95% CI)** | ***P*** | **HR (95% CI)** | ***P*** |
| All patients |  |  |  |  |  |
| 6 - month therapy group | 51 (88.7) | 1 |  | 1 |  |
| 3 - month therapy group | 51 (68.7) | 3.23 (1.13-9.21) | 0.028 | 4.35 (1.46-13.00) | 0.008 |
| Stage III |  |  |  |  |  |
| 6 - month therapy group | 35 (90.3) | 1 |  | 1 |  |
| 3 - month therapy group | 36 (64.5) | 4.22 (1.13-15.73) | 0.032 | 5.88 (1.44-24.04) | 0.014 |
| High-risk Stage II |  |  |  |  |  |
| 6 - month therapy group | 16 (83.3) | 1 |  | 1 |  |
| 3 - month therapy group | 15 (74.6) | 1.78 (0.30-10.66) | 0.529 | 3.58 (0.44-28.80) | 0.231 |

Abbreviation: CI, confidence interval; DFS, disease-free survival; HR, hazard ratio.

* Multivariate analysis adjusted for age, pathologic T stage, pathologic N stage, initial bowel obstruction, vascular invasion and/or lymphatic infiltration, and perineural invasion.
