# Supplementary material for: Duration of FOLFOX Adjuvant Chemotherapy in High-Risk Stage II and Stage III Colon Cancer With Deficient Mismatch Repair
Source: Front Oncol. 2020 Dec 4;10:579478. doi: 10.3389/fonc.2020.579478 (PMC7747753; doi:10.3389/fonc.2020.579478)
Supplement: Supplementary file 3 [file Table_3.docx]

| Supplementary Table S3. Analysis of disease-free survival (DFS) between patients in 6 -month therapy group and surgery alone group after propensity score matching. | | | | | |
| --- | --- | --- | --- | --- | --- |
| **Variable** | **No. of patients (3-year DFS rate, %)** | **DFS** | | | |
|  |  | **Univariate analysis** | | **Multivariate analysis ^*^** | |
|  |  | **HR (95% CI)** | ***P*** | **HR (95% CI)** | ***P*** |
| All patients |  |  |  |  |  |
| 6 - month therapy group | 35 (84.6%) | 1 |  | 1 |  |
| Surgery alone group | 35 (65.4%) | 2.63 (0.91-7.59) | 0.074 | 3.02 (1.00-9.07) | 0.049 |
| Stage III |  |  |  |  |  |
| 6 - month therapy group | 21 (85.4) | 1 |  | 1 |  |
| Surgery alone group | 21 (59.2) | 2.70 (0.69-10.56) | 0.152 | 3.25 (0.78-13.54) | 0.106 |
| High-risk Stage II |  |  |  |  |  |
| 6 - month therapy group | 14 (82.5) | 1 |  | 1 |  |
| Surgery alone group | 14 (73.1) | 2.38 (0.42-13.53) | 0.328 | 2.30 (0.36-14.87) | 0.383 |

Abbreviation: CI, confidence interval; DFS, disease-free survival; HR, hazard ratio.

* Multivariate analysis adjusted for age, pathologic T stage, pathologic N stage, initial bowel obstruction, vascular invasion and/or lymphatic infiltration, and perineural invasion.
